# Lipidomics from sample preparation to data analysis: a primer

**DOI:** 10.1007/s00216-019-02241-y

**Published:** 2019-12-10

**Authors:** Thomas Züllig, Martin Trötzmüller, Harald C. Köfeler

**Affiliations:** Core Facility Mass Spectrometry, Medical University of Graz, Stiftingtalstrasse 24, 8010 Graz, Austria

**Keywords:** Lipidomics, Mass spectrometry, Chromatography, LC-MS, Shotgun lipidomics

## Abstract

Lipids are amongst the most important organic compounds in living organisms, where they serve as building blocks for cellular membranes as well as energy storage and signaling molecules. Lipidomics is the science of the large-scale determination of individual lipid species, and the underlying analytical technology that is used to identify and quantify the lipidome is generally mass spectrometry (MS). This review article provides an overview of the crucial steps in MS-based lipidomics workflows, including sample preparation, either liquid–liquid or solid-phase extraction, derivatization, chromatography, ion-mobility spectrometry, MS, and data processing by various software packages. The associated concepts are discussed from a technical perspective as well as in terms of their application. Furthermore, this article sheds light on recent advances in the technology used in this field and its current limitations. Particular emphasis is placed on data quality assurance and adequate data reporting; some of the most common pitfalls in lipidomics are discussed, along with how to circumvent them.

## Introduction

Lipids are an important class of biomolecules that are involved in many vital cellular processes. Due to their hydrophobic nature, lipids are the major constituents of biological membranes and are thus the physical basis of all living organisms because they provide the ability to separate living entities from their natural surroundings. Another task that lipids fulfil is the storage of surplus energy for later consumption. Finally, lipids are also involved in extra- and intracellular signaling processes, where they transduce signals and amplify regulatory cascades.

**Fig. 1:**
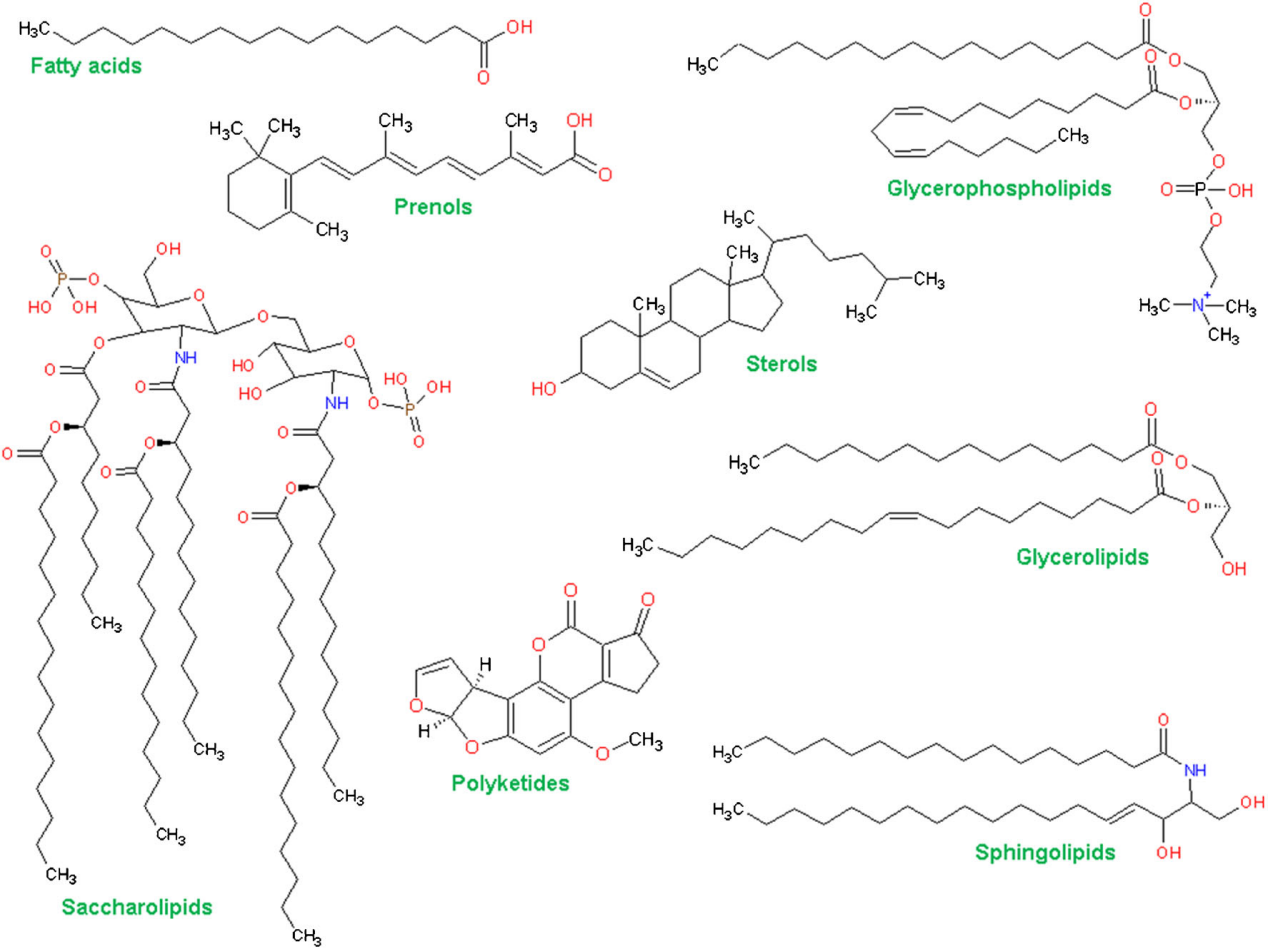
Lipid categories according to the International Lipids Classification and Nomenclature Committee, with one representative structure shown for each category

From a chemical point of view, lipids are a heterogeneous pool of compounds that all contain either fatty acyl/alkyl, sphingosine, or isoprene moieties as their hydrophobic building blocks. Since 2005, lipids have been classified into eight categories: (1) fatty acyls, (2) glycerolipids, (3) glycerophospholipids, (4) sphingolipids, (5) sterols, (6) prenol lipids, (7) saccharolipids, and (8) polyketides (Fig. 1) [1]. Each of these eight lipid categories consists of further lipid classes and subclasses, leading to a total of 43,636 lipids in the LIPID MAPS Structure Database (LMSD), among which 21,683 compounds are curated and 21,953 are computationally generated lipids (October 2019).

**Fig. 2:**
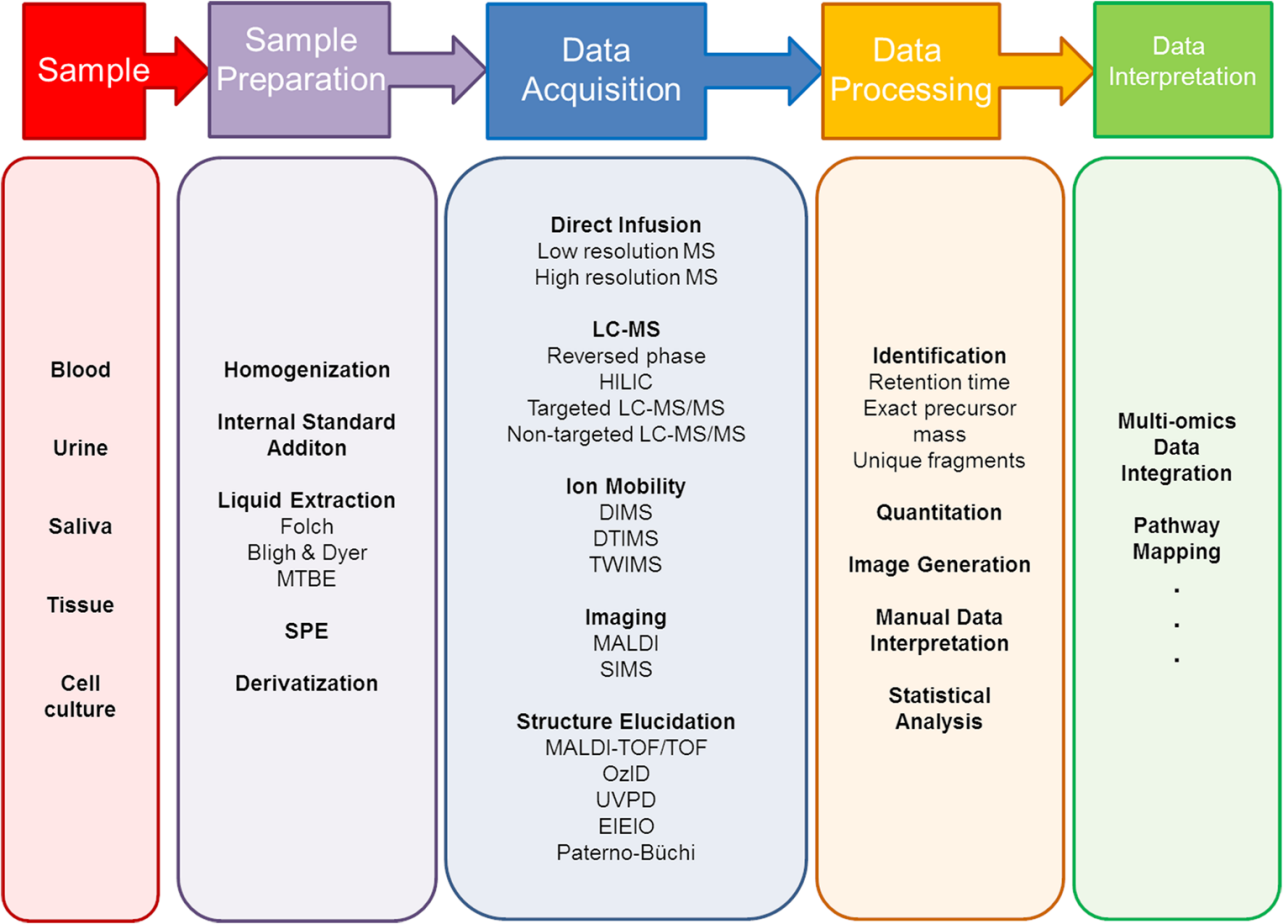
The lipidomics workflow, including all essential steps from sample to biological outcome

Since lipids play a crucial role in many biological processes, any imbalance in their homeostasis can lead to serious conditions in living organisms, such as chronic inflammation, cardiovascular diseases, diabetes, and neurodegenerative diseases, to name just a few. Hence, the importance of identifying and quantifying lipids in biomedical research should not be underestimated. The method of choice for the analysis of lipid molecules or huge assemblies of them (known as the* lipidome*) is undoubtedly mass spectrometry (MS), due to its sensitivity and specificity [2]. Because of the inherent chemical complexity of the lipidome and the consequent challenges associated with analyzing it, progress in the field of lipidomics lagged behind the progress made in other omics disciplines for a long time. However, within the last decade, the output of publications on lipidomics has increased by a factor of 7.7 according to Web of Science, which makes it one of the fastest-growing research fields of the last decade. As depicted in Fig. 2, the workflow of a typical lipidomics analysis comprises sample preparation, data acquisition, data processing, and data interpretation. In this review, we will discuss the analytical methods and workflows that have led to the tremendous expansion of the field of lipodomics, and we will also introduce and critique the technical challenges and limitations of these methods.

## Sample processing

The first and most vital step in sample processing is the sampling itself. If samples are not immediately processed or flash frozen, many enzymatic and chemical processes will continue, eventually metabolizing the lipids. For example, it was shown that the concentration of lysophosphatidylcholine (LPC) or lysophosphatidic acid (LPA) in plasma artificially increases when the sample is left at room temperature for too long [3, 4]. Monolysocardiolipin, on the other hand, is thought to be generated by the hydrolysis of cardiolipin (CL) during the freezing process [5]. When samples are left in methanol at temperatures above 20 °C and at pH > 6, lysophospholipid regioisomers will start to isomerize until they reach equilibrium [6]. In general, particular care needs to be taken whenever any degradation or oxidation products of lipids such as oxidized phospholipids, oxylipins, and lysolipids need to be analyzed, and even more so when their concentrations are expected to be very low, which is very often the case. Therefore, it is highly advisable to process samples as quickly as possible, or, if this is not possible, to store them at least at −80 °C. Since lipids are generally prone to oxidization and hydrolysis, it is advisable to restrict the storage of samples to a period that is as short as possible, even at −80 °C.

### Sample homogenization

While homogenization is not a big issue for biofluids such as serum, plasma, or urine, this step becomes very important when working on tissue samples or cells. Homogenization ensures that lipids from all parts of a piece of tissue are equally accessible to extraction solvents. For cells, it is important to rupture the cell wall in order to obtain full access to intracellular domains (e.g., organelles). Limited solvent accessibility of tissues or cells can result in significantly distorted lipid profiles (unpublished observation relating to mouse liver). Frequently used homogenization methods are the shear-force-based grinding of tissue (Potter–Elvehjem homogenizer, ULTRA-TURRAX) in a solvent or the crushing of liquid-nitrogen-frozen tissue by pestle and mortar [7, 8]. Since the latter approach is performed manually, it is rather slow. Additionally, frozen tissue pieces often contain some ice, which could distort the results when they are normalized based on frozen tissue weight [7]. Cells can be disrupted by a pebble mill with beads or a nitrogen cavitation bomb [7, 9]. While the first method disrupts cell walls by applying shear force (the cells are beaten by specialized Lysing Matrix beads), the second method combines high pressure (of inert gaseous nitrogen) with subsequent fast adiabatic expansion, thus avoiding any shear stress to the biomolecules.

### Liquid–liquid extraction

Any extraction method used in lipidomics serves two main purposes. First of all, it reduces the complexity of the sample by getting rid of any unwanted nonlipid compounds. Since this can also be seen as a method of reducing levels of contaminants, a positive side effect of sample extraction is a less contaminated mass spectrometer, which in turn leads to less instrument downtime due to maintenance and cleaning. The second aim of sample preparation is to enrich the analytes of interest (in our case lipids), leading to improved signal-to-noise ratios.

The sample preparation technique most widely used in lipidomics is liquid–liquid extraction. The Folch protocol and the Bligh and Dyer protocol both rely on a ternary mixture of chloroform, methanol, and water [10, 11]. In this setting, the organic chloroform/methanol phase contains the lipids and the aqueous phase contains the more hydrophilic compounds and salts. For anionic lipids such as phosphatidic acids (PA), LPA, phosphatidylinositols (PI), or sphingosine-1-phosphate (S1P), it is advisable to modify this protocol by adding some acid to neutralize these anionic lipids and thus improve solubility in the organic phase, which then results in increased extraction efficiency. However, when using an acidic extraction protocol of this nature, it is highly recommended that the acid concentration and extraction time should be strictly controlled; otherwise, hydrolysis artefacts such as lysophospholipids are easily generated, leading to false-positive data in the worst case scenario [12]. A variant of chloroform-based extraction is the alkaline hydrolysis protocol. In this, the addition of a 0.2 M sodium hydroxide solution to the aqueous phase hydrolyzes all of the ester bonds but not the amide bonds of sphingolipids. After neutralization by acetic acid, this protocol yields a lipid extract devoid of glycerophospholipids, glycerolipids, and sterol esters, leaving only sphingolipids intact for subsequent analysis in mammalian samples. This in turn greatly enhances the detectability of sphingolipids, which would be suppressed to a greater degree by more abundant lipid classes such as phosphatidylcholine (PC) in a nonhydrolyzed extract. In contrast to the aforementioned liquid–liquid extraction protocols, the extraction method proposed by Matyash et al. is based on methyl *tert*-butyl ether (MTBE) instead of chloroform [13]. Upon mixing MTBE with methanol and water in the ratio 5:1.5:1.25 (v/v/v), the upper organic phase retains the lipids while salts and hydrophilic compounds are enriched in the lower aqueous phase. The extraction efficiency is comparable to the Bligh and Dyer protocol, but handling is much easier since the upper layer (MTBE) is removed more efficiently by pipetting than the lower layer (chloroform). Additionally, MTBE is less harmful than chloroform, which is an important safety aspect for people exposed to these chemicals on a daily basis. A recently published comparison of chloroform and MTBE protocols suggests that the MTBE method is more efficient for glycerophospholipids, ceramides, and unsaturated fatty acids, while the chloroform protocol is superior for saturated fatty acids and plasmalogens [14]. Recently, an interesting one-step extraction protocol for mouse plasma was published by Satomi et al. [15]. The authors compare methanol, ethanol, 2-propanol, and acetonitrile to the MTBE method described above. These procedures all involve the addition of an organic solvent and the spinning down of the precipitated proteins. Obviously, these protocols result in an increased number of nonlipid compounds in the extract compared to two-phase systems. This may lead to increased instrument contamination and greater ion suppression effects. That said, one-step protocols are fast and robust and show higher extraction efficiencies for polar lipids than the MTBE method. This effect is particularly pronounced for LPC, lysophosphatidylinositols (LPI), gangliosides, bile acids, acylcarnitines, and S1P. The BUME (butanol/methanol) method is a completely automated extraction protocol that is based on the sequential addition of butanol/methanol (3:1), heptane/ethylacetate (3:1), and finally acetic acid for phase partitioning [16]. Just as in the MTBE protocol, the organic phase constitutes the upper layer. This method has comparable extraction efficiency to the Folch protocol, but can be fully automated for high-throughput screening in 96-well plates and avoids the need for hazardous chloroform. A recently published three-phase lipid extraction protocol relies on the addition of hexane, methyl acetate, acetonitrile, and water, which results in three distinct phases: a lower aqueous phase, an organic phase in the middle, and an upper organic phase [17]. While the middle organic phase contains mostly polar lipids (e.g., glycerophospholipids, sphingolipids), the upper organic phase contains mostly neutral lipids (e.g., triacylglycerols (TG)). Both organic phases can then be acquired in separate analytical runs. Finally, the increased separation of lipids is reflected in an increased identification rate due to the reduced sample complexity in each run.

### Solid-phase extraction

Solid-phase extraction (SPE) is a very specific sample preparation technique used in lipidomics that yields highly enriched samples with little contamination. On the downside, extraction protocols become highly sophisticated and laborious as the number of lipid classes to be analyzed increases. Thus, SPE is clearly not the method of choice if many lipid classes need to be analyzed in a high-throughput fashion, but it can be a highly valuable method if only few precious samples need to be analyzed with a very high coverage of lipid species. For instance, Fauland et al. proposed a separation scheme that utilizes three different SPE cartridges and six eluents and results in seven fractions at the end of the fractionation process [18]. Another example of the usefulness of SPE is the separation of gangliosides from the aqueous phase of a Folch extract [19]. Since this is achieved using a C-18 SPE cartridge, gangliosides are selectively separated from other polar compounds by their ceramide moieties after other nonpolar lipids have already been removed by the previous Folch extraction. This ultimately results in a highly ganglioside-enriched fraction of the sample.

### Derivatization

Derivatization serves four purposes in lipidomics: it can be used to (1) increase ionization efficiency, (2) introduce a selective fragment that can even be used in precursor ion or neutral loss scans, (3) mask functional groups (e.g., phosphates) that may stick to steel surfaces when they are transferred to the mass spectrometer, and (4) introduce an isotopic label for differential quantitation. A good example of the first category is the reaction of Girard’s Reagent P with the hydroxy group at C3 in sterols and oxysterols [20]. In this case, the C3 hydroxy group is first oxidized to a keto group, after which it reacts with Girard’s Reagent P to form a hydrazone and to introduce a quaternary nitrogen atom, which greatly enhances the ionization properties of the molecules of interest in positive electrospray ionization. The second strategy is illustrated by a recent publication from the group of Han, who used phosphate group methylation of phosphatidylglycerols (PG) and bis(monoacylglycero)phosphates (BMP) [21]. This diazomethane-based methylation introduces class-specific fragments into the MS/MS spectra of PG and BMP, which yield very similar fragmentation patterns without derivatization. A paper published by Clark et al. also utilizes the methylation of phosphate groups (from phosphatidyl inositol phosphates, PIP), but in this case the main benefit of methylation is the masking of free phosphates, which enables better quantitative transfer of minute amounts of PIP from the HPLC via connective tubes to the mass spectrometer and enhances ionization efficiency in positive electrospray ionization (ESI) [22]. The fourth strategy aims at the introduction of a stable isotope-labeled derivatization moiety into one sample, whereas the comparison sample receives an unlabeled derivatization moiety. A good example of such a strategy was demonstrated by Lee et al. [23], who methylated glycerophospholipids at their phosphate groups using (trimethylsilyl)diazomethane. Since this reaction was carried out once in HCl/MeOH and once in DCl/MeOD, the introduced methyl group contained either two H atoms or two D atoms, which resulted in a mass shift of 2 Da for each methyl group. By mixing together equal amounts of the samples, it was possible to directly compare the labeled and unlabeled signals from the same lipids and determine the ratio of each lipid in a direct comparison between both samples. This application was shown to work for PA, PI, PIP, PS, PG, and CL and is an elegant approach for the direct comparison of samples.

## Direct infusion lipidomics

The term ‘shotgun lipidomics,’ which refers to direct-infusion ESI mass spectrometry without any prior separation technique, was coined about 20 years ago by the group of Han, who was in fact the first scientist to utilize electrospray for lipidomics in 1994 [24]. The basic concept of these triple-quadrupole-based methods involves the selective ionization enhancement of certain lipid classes, which is termed intrasource separation, and the subsequent application of precursor ion and neutral loss scans of polar head group and fatty acid moieties [25]. In terms of identification, this approach exclusively relies on a set of unique fragments for each lipid. A variant of this setup is flow injection analysis, which was proposed by Liebisch in the late 1990s and has since been applied to most major mammalian lipid classes [3, 26]. Flow injection analysis uses a HPLC without a chromatographic column, with the sample injected into a continuous isocratic flow of mobile phase as an unseparated peak. All of the required single reaction monitoring (SRM), precursor ion, and neutral loss scans have to be performed during this peak. The advantage of this method over direct infusion via syringe is that it can be automated via a HPLC autosampler. For both of these infusion setups, carryover effects can be a problem and must be closely monitored for in order to achieve high data quality. A good solution to this issue is the Nanomate nano-ESI chip developed by Advion Inc. (Ithaca, NY, USA), which relies on one nanoESI spray needle for each sample and therefore eliminates any carryover from the injection system [27]. Furthermore, nano-ESI greatly enhances signal intensities, resulting in better detection limits, and it minimizes the amount of sample needed [28]. Thus, a nano-ESI chip is considered a contemporary and highly useful piece of equipment when performing shotgun lipidomics. The major strength of any shotgun lipidomics approach when compared to chromatography-based methods is its steady ionization environment, which results in a very robust basis for quantification. Thus, it is possible to get quantitative results with just one nonendogenous internal standard per lipid class, which would be impossible to achieve with most chromatographic settings [25, 27, 29]. This quantitative stability is corroborated by a long-term study on human plasma, where the obtained concentrations were found to be remarkably robust and reproducible over 3.5 years. The coefficients of variation of the mean lipid concentrations were mostly below 15%, meaning that shotgun lipidomics would even comply with Food and Drug Administration (FDA) requirements according to good laboratory practice (GLP). On the downside, shotgun lipidomics is prone to ion suppression effects due to the inherently limited ionization capacities in the electrospray process, which, in the worst case scenario, may even completely suppress signals from minor lipid constituents with poor ionization properties. In that case, a custom chromatography-coupled approach may be the method of choice instead. Another challenge associated with nano-ESI is the limited stability of the electrospray due to clogging. Furthermore, shotgun lipidomics lacks the additional analytical dimension introduced by any separation technique, and so also lacks some of the identification certainty inherent to liquid chromatography–mass spectrometry (LC-MS) approaches. The lack of chromatographic separation is also a disadvantage when working with complex samples with lots of isobaric/isomeric lipids. For example, it is not possible to separate PCs with odd-carbon-numbered fatty acids from plasmalogens if they are isobaric [30]. To deal with such uncertainty, many users have transferred from triple-quadrupole instruments to high-resolution mass spectrometers with quadrupole–time of flight (Q-TOF) or Orbitrap technology [27, 29, 31]. While Q-TOF-based technology offers the mass resolution of 40,000 needed to separate the above-described isobaric example of plasmalogens and diacyl PC species, the mass resolution required to separate ^13^C, ^17^O, ^2^H, or ^15^N isotopologues from either each other or other monoisotopic lipid species is above 100,000, and can only be provided by Orbitrap or ion cyclotron technology. Nevertheless, in natural samples, all minor isotopes except for ^13^C can be neglected for practical purposes due to their low abundances, but it is highly beneficial to separate the M + 2 isotopic peak of a lipid, which is mainly due to ^13^C, from the monoisotopic mass peak of the same lipid with one double bond less. A resolution of 200,000 full width at half height is sufficient to separate these overlapping isotopes. For more information on the correlation between mass resolution and identification ambiguities, readers are referred to Bielow et al., who cover this topic in depth [32]. Instrumentation with a mass resolution beyond 500,000 makes it possible to perform isotopic labeling experiments and subsequent metabolic flux analysis with isotopes that have naturally low abundances, such as the technique proposed by Schuhmann et al. for ^15^N labeling in human HepG2 cells [33]. A recently published and interesting approach to addressing the ion suppression challenges arising from direct infusion is spectral stitching [33, 34]. The proposed acquisition protocol parses the mass range of the full-scan spectrum into smaller selected ion monitoring (SIM) mass ranges of between 20 and 50 Da, which are acquired sequentially and then ‘stitched’ together by the software into one large full-scan spectrum. Although ion suppression effects arising from the ESI process are still an issue with this method, it does alleviate ion suppression effects arising from the inherently limited fill capacity and dynamic range of Orbitrap and Fourier transform–ion cyclotron resonance (FT-ICR) analyzers. In contrast to chromatography-coupled methods, the acquisition time in nano-ESI chip-based shotgun lipidomics is virtually limited only by the sample volume, which can result in an acquisition time of more than 30 min per sample. This facilitates techniques such as data-independent acquisition (DIA) MS/MS^ALL^ with very fast high-resolution instruments such as a TripleTOF, and provides MS/MS spectra for all masses with an isolation width of 1 Da across a mass range as wide as 1000 Da [35]. This method has the advantage that no information is lost, and MS/MS spectral coverage is 100% over a wide mass range. Furthermore, it was shown that MS/MS^ALL^ can be performed in a quantitative manner, and the method was successfully applied for the quantification of CL in mitochondrial preparations. A disadvantage of MS/MS^ALL^ is the eventual loss of information on precursor–fragment relationships, which potentially complicates the identification of lipid species. The MS^ALL^ approach published by Almeida et al. relies on the same concept but also involves the acquisition of full-scan spectra at a resolution of 450,000 (*m/z* 200), split into low and high *m/z* ranges with positive and negative polarity [31]. Furthermore, it utilizes the full fragmentation power of an Orbitrap Fusion Tribrid, and sequentially acquires higher-energy collisional induced dissociation (HCD) and collisional induced dissociation (CID) MS/MS spectra at* R * = 30,000 (*m/z* 200) as well as CID MS^3^ spectra for specific compounds. Using these complementary analytical tools, it was possible to quantify 311 lipid species in mouse cerebellum and hippocampus and to explore the structures of 202 of these lipids in depth [31].

In recent years, desorption electrospray ionization (DESI) has become a noteworthy option for direct infusion lipidomics. In a comparative study with LC-MS, it was shown that DESI-MS forms different adducts than LC-MS, but when adjusted for these different adducts, the mass spectra show a very high degree of correlation in the determined lipid composition [36]. The major advantage of DESI in lipidomics is the ability to use it for mass spectrometry imaging (MSI). This was shown nicely for various tissues from mammalian sources by Klein et al. [37].

## LC-MS

The chromatographic separation method most widely used in lipidomics is reversed-phase HPLC, which separates lipids based on their nonpolar fatty acyl moieties [38]. The underlying mechanism is described by the equivalent carbon number theory. According to this model, lipids of the same class are separated according to a combination of their cumulative number of fatty acyl carbons and the number of fatty acyl double bonds, as retention time increases as the number of carbons increases and decreases as the number of double bonds increases. It was recently reported that the use of a reversed-phase nano-HPLC setup increased the number of detected lipids by a factor of 3.4 when compared to the more frequently used narrow-bore HPLC setup [39]. In contrast to reversed-phase HPLC, normal-phase chromatography separates lipids based on their polar head groups, but from a practical perspective, normal-phase HPLC is poorly compatible with the ESI process of ion formation due to its extremely high nonpolar organic solvent content. Nevertheless, highly specialized normal-phase HPLC techniques such as silver ion chiral chromatography coupled to atmospheric pressure chemical ionization (APCI) can be applied to achieve a detailed analysis of TGs, including the positions of fatty acyls and even the locations and geometries of double bonds [40]. Hydrophilic interaction liquid chromatography (HILIC) also resolves lipids according to their polar head groups, but the solvents used are much less hydrophobic and are thus much more compatible with ESI. Therefore, HILIC is also frequently used in lipidomics [19, 41, 42]. In a HILIC run, all species of a particular lipid class elute in a very narrow time window, resulting in one unresolved chromatographic peak in which lipid species are only distinguished based on their mass. This makes quantification easier—due to the high degree of coelution of all species in a given lipid class, one internal standard with one response factor is sufficient for quantification, similar to shotgun lipidomics [43]. In comparison, reversed-phase HPLC requires many more internal standards per lipid class because the species are spread across a much wider retention time window, leading to much stronger differential ion suppression effects. This would ideally be compensated for by applying one internal standard for each compound. In reality, however, it is possible to get a good quantitative approximation by using a set of four or more internal standards for each lipid class, which should ideally be spread over the whole retention time range of the class [44]. On the other hand, reversed-phase HPLC is able to separate isomeric lipid species and therefore offers the potential for in-depth structural elucidation [38, 45]. It should be noted that, according to the Web of Knowledge, ultra-high performance liquid chromatography (UHPLC) has increasingly replaced conventional HPLC systems over the last decade because UHPLC provides faster run times at higher chromatographic resolutions due to its much higher backpressure allowance. The challenge with ever-decreasing peak widths is the duty cycle time of the mass spectrometer, which still has to allow for a sufficient number of sampling points per chromatographic peak. Therefore, instruments that perform rapid scans, such as q-TOFs, are the preferred choice for fast UHPLC-delivered gradients with narrow peak widths.

**Fig. 3:**
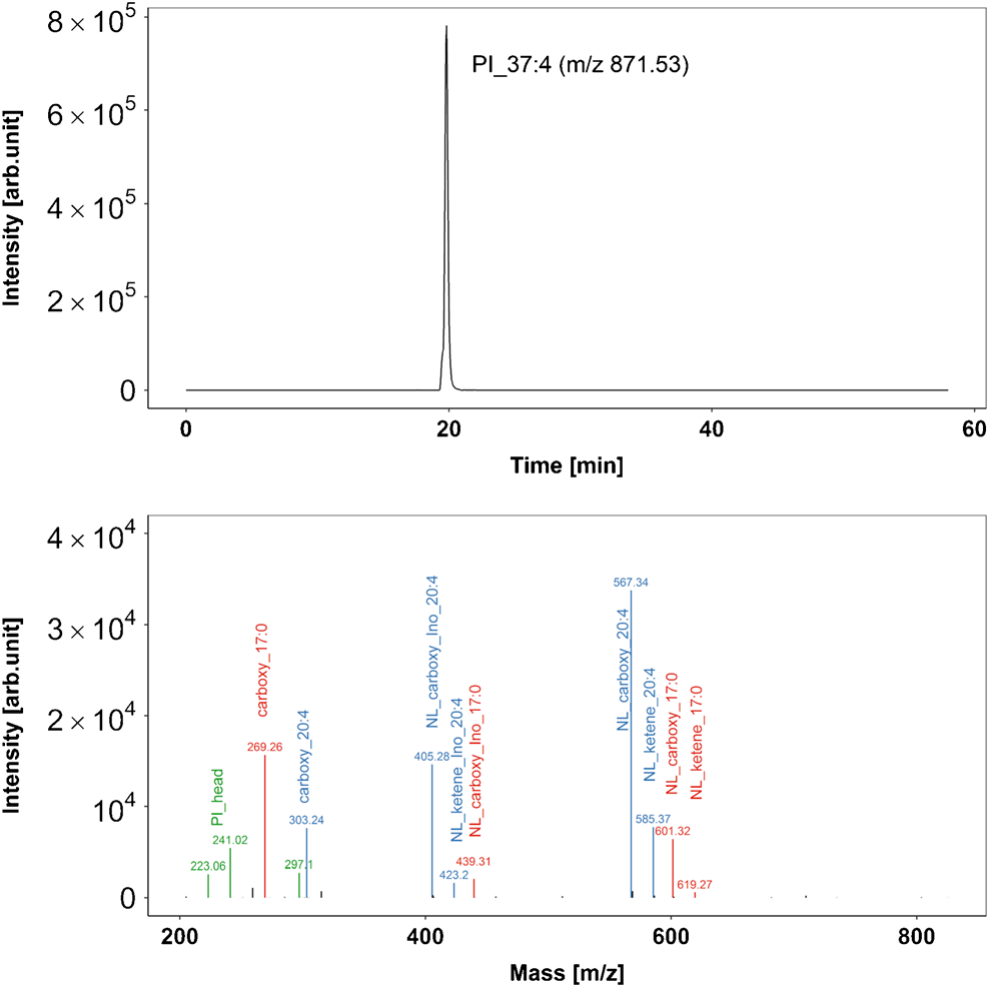
The* upper panel* shows an extracted-ion chromatogram of glycerophosphoinositol, PI 37:4 (*m/z* 871.534 ± 5 ppm), acquired by an Orbitrap instrument running at a resolution of 100,000 (*m/z* 400). The* lower panel *shows the corresponding MS/MS spectrum at a retention time of 19.82 min (CID fragmented). Neutral loss (*NL*) of carboxy and inositol (*Ino*) or ketene and inositol can be discerned.* Blue* fragments of 20:4 acyl chains,* red* fragments of 17:0 acyl chains,* green* fragments of PI head groups

From the perspective of scope, the coupling of HPLC with mass spectrometry can be realized using two fundamentally different approaches: targeted analysis or nontargeted analysis, with the former being easily scalable and the latter being more comprehensive. Targeted approaches are performed by applying a predefined set of compounds with known fragmentation behaviors and ideally also retention times. They can be carried out using a triple quadrupole MS in SRM mode, with just a few or sometimes even only one lipid class considered per HPLC run [46, 47]. Such a focused analysis also results in a high degree of flexibility for developing custom lipid-class-specific chromatography techniques. HILIC separation, on the other hand, leads to clearly separated lipid classes and is ideal for designing accurate time windows for SRM transitions, thus enabling more SRM transitions to be squeezed in per HPLC run [43]. Recently, parallel reaction monitoring with a Q-Exactive instrument was shown to be a alternative method with high mass resolution for the targeted determination of sterols and sphingolipids [41, 48]. Furthermore, targeted Q-TOF analysis based on full-scan high-resolution MS/MS spectra yielded the most gangliosides reported so far for biological samples [19]. In the realm of nontargeted lipidomics, high mass resolution is indispensable because increasing the mass resolution and thus the mass accuracy greatly enhances identification certainty, which is of particular interest when attempting to pin down unknown compounds. Furthermore, the availability of fragment spectra and retention times markedly improves the identification certainty (Fig. 3). Therefore, numerous high-resolution lipidomics platforms have been published, and all of them rely on data-dependent acquisition (DDA) of MS/MS spectra [49–53]. Although the described workflow is perfectly acceptable for nontargeted analysis, the data acquired in such a manner can also be subjected to a targeted analysis with predefined search lists of lipid masses and eventually even their expected fragment masses [49, 54]. Thus, it is the data processing step that dictates whether a large-scale targeted approach or even a completely nontargeted approach is adopted. The big advantage of completely nontargeted lipidomics is that it is able to potentially highlight every compound in a sample and thus discover hitherto unknown lipid species. The biggest limitations on the comprehensiveness in this regard are the restricted capacity for ionization, which is particularly true of ESI and ion storing analyzers, as well as the suitability of the ionization technique, where tradeoffs have to be made because not every ionization technique is equally well suited to every lipid class. Another limitation is lipid extraction, which inherently results in a bias and may even exclude some lipid classes. These limitations may be alleviated by using, for example, multiple extraction methods for polar, nonpolar, and acidic lipids, which are then acquired by ESI and APCI in positive and negative polarity modes, and even split mass ranges, but the effort required also increases dramatically when 12 or more LC-MS runs per sample are needed to ensure comprehensiveness. The good news in this respect is that a comparison of the results from seven Q-TOF models, a Q-Exactive, and a single time-of-flight (TOF) mass spectrometer showed that there was no significant difference between these platforms, suggesting that the resulting up- and downregulation patterns do not seem to be greatly influenced by the choice of high-resolution mass spectrometer [53]. The big challenge with every nontargeted approach is the step from mere features to chemically and biologically meaningful entities [55]. Although the beauty of nontargeted setups lies in their reduction of sample complexity to just a small proportion of all the features that are significantly up- and down regulated, it can still leave the mass spectrometrist with several hundred features that must be identified and often manually evaluated. Needless to say, this is a very time-consuming process that is still far from being automated. An interesting alternative to nontargeted lipidomics is the recently published concept of pseudo-targeted lipidomics [51], in which a nontargeted analysis is performed and the putative retention times and masses of lipids not detected by the nontargeted approach are extrapolated from the detected lipids. Using in silico calculations, the authors of [51] designed a multiple reaction monitoring (MRM)-based targeted method utilizing a triple quadrupole MS and involving 3377 MRM transitions in three separate LC runs, and hoped to find additional previously undetected lipids in their sample. This resulted in an increase from 494 to 823 detected lipid species in mouse serum. By the same token, the concept of iterative exclusion omics involves performing multiple injections of the same sample to expand its MS/MS coverage in DDA mode [56]. This results in a significantly increased lipid identification rate, and is particularly useful in densely crowded elution ranges (e.g., the glycerophospholipid region and the TG region).

### Supercritical fluid chromatography

In recent years, supercritical fluid chromatography (SFC) has become a promising branch of chromatography used in the field of lipidomics. The basic merits of SFC over conventional HPLC are its faster elution times and higher chromatographic resolution. For a long time, the compatibility of the mobile phase (supercritical carbon dioxide) with ESI posed a major issue from a robustness standpoint, but the addition of a compatible make-up liquid after the column greatly improved the suitability of SFC for ESI [42, 57, 58]. For example, an ultra-high performance SFC (UHPSFC) equipped with a HILIC column was able to separate 25 lipid classes in a run time of just 6 min [57]. When this method was compared to UHPLC, it became evident that UHPSFC was able to identify 3.4 times more lipid species in a chromatographic run time that was 40% shorter [42]. In a similar approach, Takeda et al. showed that it is even possible to separate positional isomers of lysoglycerophospholipids and monoacylglycerol and diacylglycerol species on a diethylamino column when the runtime was increased to 18 min [58].

## Quantitative aspects

Great care needs to be taken when quantifying lipids by ESI-MS because, compared to nuclear magnetic resonance spectroscopy (NMR), UV or flame-ionization (FID) ESI-MS detection is much less scalable for a variety of reasons, including the competitive nature of the ESI process and the isotopic distribution and mass dependence of the fragmentation patterns [59]. All of these obstacles can be circumvented by using stable isotope-labeled internal standards that differ from the target compounds in their physical properties (i.e., mass) but not their chemical properties (retention, ionization, fragmentation). In a perfect scenario, each target lipid would be referred to just one internal standard via a calibration curve that covers the expected concentration range. However, the feasibility of this approach is limited by the availability of the internal standards, the costs involved, and the practicability of running hundreds or even thousands of calibration curves for each sample batch. Due to these limitations, just a handful of internal standards are usually employed in lipidomics, making the selection of appropriate internal standards a very important task [60]. Recently, commercial mixes of internal standards tailored to individual body fluids, tissues, and organisms or intended for other purposes have become available. These mixes contain internal standards in the expected concentration ranges of the lipid classes typically found in the biological matrix of interest. In shotgun lipidomics of polar lipids (e.g., glycerophospholipids), it is possible to achieve fairly accurate quantification using just one internal standard per lipid class so long as the quantification is done in a MS1 survey scan by high-resolution instruments. This is because ionization efficiency is dependent only on the polar head group (not the fatty acyl tails) if the concentration of lipids is not too high (avoiding aggregation). When precursor ion or neutral loss scans are included in the quantification process, one internal standard per lipid class is still sufficient as long as the mass dependence of the fragmentation is circumvented by gradually increasing the collision energy with increasing *m/z* of the precursor ion [60]. If the collision energy is not increased, at least two internal standards and a correction function are necessary [61]. Since neutral lipids such as TGs do not have a polar head group to localize the charge, the dependence of the ESI efficiency on the fatty acyl composition is more pronounced, so more than one internal standard is needed per lipid class to compensate for discriminatory ionization effects [60]. When performing LC-MS-based lipidomics, at least four internal standards per lipid class, ideally distributed across the whole elution time range, are needed; even then, the data obtained will be less accurate than that attained using a shotgun approach [44]. If the user is only interested in a limited number of lipid species, a SRM method with high internal standard coverage and extensive validation is of course the best choice [62]. Hence, unless the user has unlimited resources, it is always highly advisable for them to figure out in advance what the scope should be and which lipid species or lipid classes are really of interest, as narrowing the focus will make the quantitative aspects more reliable. A solution worth considering in this respect is a standardization method developed within the last decade called lipidome isotope labeling of yeast (LILY), which provides as many as 212 ^13^C-labeled internal standards [63]. In brief, *Pichia pastoris* is grown on ^13^C-enriched cell culture medium, the almost fully ^13^C-labeled lipid content is extracted, and the labeled lipidome is then used as an internal standard mix for unlabeled lipids. Since there are more than 200 internal standard lipids in such an extract, very high internal standard coverage is achieved, which is particularly useful for LC-MS based lipidomics. The drawback of this method is that in order to accurately quantify all of the ^13^C-labeled lipids in the standard mixture, they still need to be compared with known amounts of their unlabeled analogs, meaning that known amounts of all of the unlabeled reference standards must be purchased. Aside from compensating for the discriminating factors during ionization and fragmentation (as discussed above), isotopic correction and the summation of all possible isotopes of a compound are important steps to ensure quantitative data [60]. When an instrument with a mass resolution of below 250,000 is used or the chromatographic resolution is insufficient, isotopic correction is needed to separate the monoisotopic and M + 2 mass peaks (–^13^C_2_H_2_– vs –^12^C_2_H_4_– moieties) of lipids that differ by only one double bond. This mathematical correction results in accurate values for both components of these mass peaks. After this processing step, it is important to sum all the isotopologues of a lipid species. From a practical perspective, only the ^13^C isotopic distribution needs to be considered, as all the other possible isotopes have very low natural abundances and therefore do not significantly influence the quantitative data.

## Ion mobility spectrometry

Since ion mobility spectrometry (IMS) separation is based on an alternative physicochemical concept, it constitutes a valuable complementary source of information when used in combination with mass spectrometry and chromatography. In a nutshell, in this technique, ions are moved by an electric field against the flow direction of an inert drift gas, resulting in ion separation based on their sizes and shapes. In contrast to size, shape is not correlated with mass, so this introduces an additional separation dimension. Whereas differential ion mobility spectrometry (DIMS) can be regarded as an additional ion filter that selects only one type of ion at a time and where the compensation voltage is scanned, drift time ion mobility spectrometry (DTIMS) produces drift-time-separated ion signals, much like a TOF analyzer. When used in combination with a shotgun approach, DIMS is able to separate lipid classes according to their compensation voltage, resulting in less complex MS/MS spectra for isobaric or isomeric overlapping lipids such as PE 38:4 and PC-O 36:4 at *m/z* 768.6 [64]. Besides its ability to separate isomeric and isobaric species and thus reduce the complexity of fragment spectra, the biggest asset of IMS is its introduction of collision cross-sections (CCS) as an additional complementary means of identification. The CCS value is a measure of the shape- and size-dependent mobility of an ion in IMS and is unique to every compound. In recent years there have been major efforts to establish lipid CCS databases for DTIMS and traveling wave IMS [65–69]. Blazenovic et al., for example, proposed a system of lipid annotation derived from accurate mass, retention time, CCS, and degree of unsaturation values as well as the number of carbons, which should promote the identification certainty for lipids from untargeted workflows [65]. In a similar fashion, Leaptrot et al. introduced an experimentally derived database of CCS values of glycerophospholipids and sphingolipids [67]. Their findings suggest that the main determinant of the CCS value is not fatty acyl chain length but rather the degree of fatty acyl unsaturation. For readers interested in the actual IMS workflows, Paglia et al. provide very detailed protocols on various IMS-coupled mass spectrometry methods [69]. Another good example of the integration of IMS into LC-coupled systems is shown by Hinz et al., who separated oxylipins and fatty acids using both chromatography and IMS [70]. Since oxylipins show a particularly high degree of isomerism, the acquisition of drift times can be a helpful tool which may even enable the positions of hydroxy groups on the fatty acyl chain to be determined. In addition to databases with experimentally determined CCS values, there are also tools such as the Lipid CCS Predictor for the in silico prediction of CCS values based on SMILES structures [66, 71]. The Lipid CCS database currently contains over 15,000 lipids with over 60,000 corresponding CCS values determined experimentally or predicted in silico.

## MALDI-TOF and mass spectrometric imaging

Although the use of matrix-assisted laser desorption–time of flight (MALDI-TOF) as a fast and robust analysis method for lipids dates back to the late 1990s, it is still not widely used in lipidomics [72]. The reasons for this are speculated to be the absence of a chromatographic dimension, the lack of a reliable fragmentation technique (unless a MALDI-TOF/TOF mass spectrometer is used), and the limited resolution compared to Q-TOF, Orbitrap, and FT-ICR analyzers. Nevertheless, with the right choice of matrix, MALDI-TOF can quickly deliver data. Examples of matrices that have been found to be advantageous for the ionization of lipids include 2,5-dihydroxybenzoic acid, α-cyanocinnamic acid, and a mixture of 9-aminoacridine and* N*-(1-naphthyl)ethylenediamine hydrochloride [73, 74]. Perhaps the biggest advantage of using MALDI in lipidomics is its ability to provide two-dimensional images of the distributions of lipids in tissues. To this end, tissues (e.g., brain, kidney) are cryodissected into slices a few micrometers thick, placed on a MALDI target, covered with the MALDI matrix, and then scanned by the laser in two dimensions, which results in pixels a few micrometers in size, each of which represents one mass spectrum. The critical step in this process is the deposition of the MALDI matrix, which should be as homogeneous as possible and must not cause any blurring of the image to ensure that accurate lateral resolution is maintained. In recent years, the coupling of MALDI with Orbitrap analyzers has enabled higher mass resolution and the use of MS/MS spectra [75, 76]. This has facilitated the structural determination of even highly complex sulfoglycosphingolipids with up to five hexose moieties [76]. Laser capture microdissection is an interesting alternative to MALDI imaging in which micrometer pixels of tissue slices are dissected by a laser, extracted, and analyzed by shotgun lipidomics based on nano-ESI and Orbitrap technology. This permits an increased amount of time for each pixel, thus making it possible to perform various *t*-SIM and MS/MS experiments that yield detailed lipidomic profiles [77].

## Structure elucidation

When the positions and conformations of double bonds in fatty acyl moieties are of interest, the platforms described thus far are usually not suitable; more specific approaches must be employed. Based on charge remote fragmentation theory, high-energy CID yields fragmentation patterns that are indicative of the positions of double bonds in fatty acids [78]. Originally developed for sector instruments, the instrument of choice for high-energy CID is now a MALDI-TOF/TOF with a fragmentation energy of 20 keV. For example, when the charge is localized in TG with Li^+^, there are a wealth of fatty acyl fragments indicative of the double bond position and the *sn* position of the fatty acyls [79]. An as-yet unresolved drawback of MALDI-TOF/TOF instruments is the isolation window of 4 *m/z*, which makes precursor ion selection problematic for biological lipid samples. Although this kind of analysis cannot be considered a high-throughput approach due to the lack of automated spectral interpretation, it can still deliver valuable in-depth information on selected lipids. Another specialized method for localizing double bonds and separating regioisomers relies on the use of ozone as a reaction partner for double bonds [80]. The final products, obtained via ozonides and Criegee intermediates, are aldehydes and Criegee ions, which are fatty acyls truncated exactly at the location of the double bond, in a similar manner to lipid peroxidation products. While this is an elegant method of determining double-bond regioisomers, it requires an adaptation of the instrument (the introduction of ozone into the collision cell) that is not yet commercially available. The use of electron-impact excitation of organic ions (EIEIO) at a kinetic energy of 10 eV has shown promise for the determination of fatty acyl *sn* positions, fatty acyl double-bond positons, and even double-bond conformations in glycerophospholipids (including plasmalogens), glycerolipids, and sphingolipids [81]. This is achieved using a branched radiofrequency (RF) ion trap EIEIO device that delivers selective fragments for separating the aforementioned types of isomers. This technique has made it possible to pin down the exact structural compositions of various commercially available natural lipid extracts. When aliphatic double bonds react with acetone and UV light in a Paternò–Büchi reaction, the cycloaddition reaction products generated are indicative of the location of the double bond, and further fragmentation of the compound can be used to pinpoint its position. This principle was utilized for an online reactor containing an UV emitter, where it was positioned between the chromatographic column and the ion source [82]. Fragmentation of the Paternò–Büchi reaction products ultimately results in fatty acyl moieties truncated at their double-bond positions. The introduction of UV-induced photodissociation (UVPD) with a 193-nm laser as a fragmentation technique has added another promising lipodomics tool for detailed structural elucidation. It was recently shown that UVPD selectively induces the scission of bonds between vinyl groups and their allylic methylene groups in phospholipids and sphingolipids, thus allowing unambiguous assignment of double-bond positions in fatty acyls and the long chains of sphingolipids [83, 84].

## LC-MS data processing

**Fig. 4:**
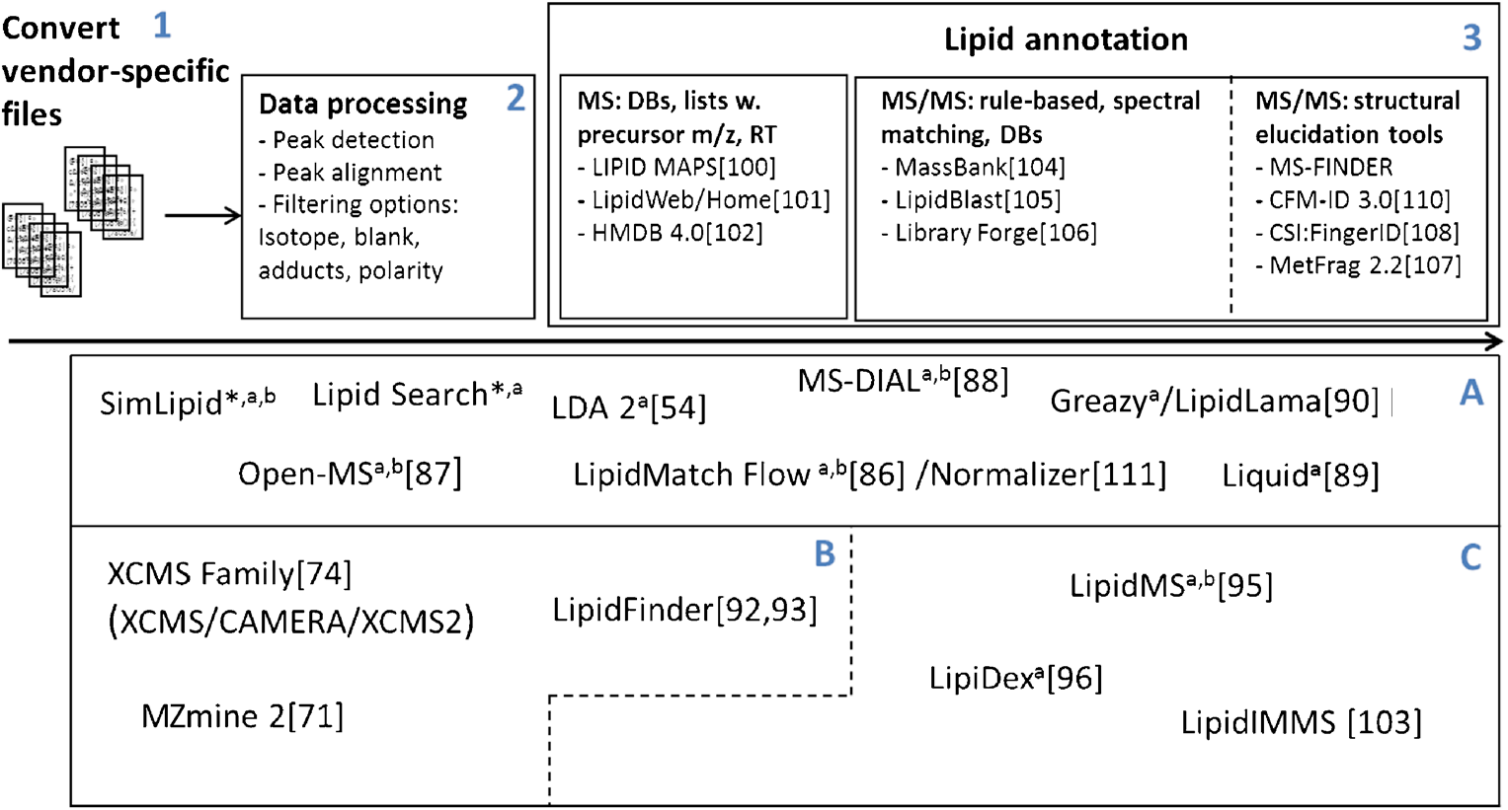
Overview of data processing and lipid annotation software grouped by functionality. Group A encompasses all-in-one software that can handle the full process pipeline: conversion of vendor-specific raw files (1); data processing, including peak detection and alignment as well as peak filtering options (2); and lipid annotation at the precursor MS level using web-based databases or precursor *m/z* lists and MS/MS derived lipid annotation at the rules-based or spectral matching level (3). Group B are software that are only used in lipidomics up to the precursor annotation stage. Group B software are used in combination with the highly specialized lipid annotation software that comprise group C. * Commercial software intended for DDA^a^ or DIA^b^ workflows

Advances in high-resolution mass spectrometry in combination with UHPLC have resulted in better structural information but also the generation of more data. Two main acquisition techniques, DDA and DIA, are used in untargeted lipidomics approaches or targeted approaches with large in-silico-based lists. In DDA workflows, instruments automatically switch from MS to MS/MS mode based on the intensity of the precursor ion and/or a precursor ion target list. One limitation of DDA approaches is that instruments are incapable of producing MS/MS fragments for all precursor ions, which can lead to undersampling, especially of low-abundance precursors. Another issue is the contamination of false MS/MS fragments due to the precursor selection window width (1 Da). There are two types of DIA strategies: (i) all-ion fragmentation (AIF), MS^ALL^, and MS^E^ approaches; (ii) sequential window acquisition of all theoretical fragment-ion spectra (SWATH). The obvious limitation of all fragmentation approaches is the lack of connection to the precursor ions, which necessitates a sophisticated data processing tool. The difference between SWATH and MS^ALL^ acquisition is that an additional isolation window of 20–50 Da is used in SWATH approaches across the entire mass range before fragmentation. This allows the fragmentation to be linked to the precursor ion because of the reduced number of interfering ions [85]. These two main approaches both generate a large amount of data. For example, a data-dependent acquisition of a MTBE-extracted serum can involve several thousand features associated with MS/MS fragments. There are various bioinformatics software tools and packages that can handle datasets of this size. Such software packages can be divided into three groups, A–C, according to their functionality (see Fig. 4). There are “all-in-one” software solutions (group A) such as Lipid Data Analyzer 2 [54] (LDA 2), LipidMatch Flow [86], OpenMS [87], MS-DIAL [88], Liquid [89], and Greazy/LipidLama [90], as well as the commercially available software packages SimLipid (PREMIER Biosoft), Lipidyzer (SCIEX), and LipidSearch (Thermo Scientific). Group B represents data processing software such as MZmine 2 [91], LipidFinder [92, 93], and the XCMS family [94] (XCMS/CAMERA/XCMS2). These are widely used to process data and generate peak tables as input files for use with group C software, which are highly specialized lipid annotation software packages such as LipidMS [95], LipiDex [96], and LipidIMMS (with an additional ion mobility dimension) [68]. Nevertheless, the interpretation of MS data to retrieve all of its structural and functional content is a challenging and evolving process.

Despite the diverse program landscape, data process pipelines are similar or even cross-functional. In a first step, vendor-specific raw files are converted into an accessible open format (e.g., mzXML, mzML, MGF, or ABF). A commonly used solution here is MSConvert from Proteowizard [97] as implemented or external tool.In this case 'implemented tool' means that it is the first step in the automated workflow of a lipidomics software package (integrated into the software), and external tool means that it also runs as stand alone file format converting software and the converted data have to be manually fed into any subsequent lipidomics software. In a second step, feature tables are generated from raw files using algorithms, retention time alignment, additional filtering, and combination options (e.g., blank filtering, isotope correction, combination of adducts and/or polarity). Many different algorithms are available that use different approaches to achieve a common goal: to find as many features as possible without incurring false positives. For example, LDA uses a 3D algorithm that depends on *m/z*, retention time, and intensity. The filtering option to verify isotopic peaks can be used to identify and remove the common false-positive M + 2 isotope, which actually corresponds to lipid species with one double bond less [98]. XCMS uses the centWave [99] algorithm a density based technique in the *m/z* domain to detect regions of interest in combination with a wavelet basedtechnique to resolve chromatographic peaks. The additional OBI-warp algorithm [94] is used to align retention time shifts. MZmine 2 [91] provides several algorithms for which users can choose and optimize parameter values within a graphical user interface (GUI) based on the quality of their MS data (e.g., peak shapes, mass resolution, or background noise). LipidMatch [86] uses MZmine 2 for data processing and offers blank filtering as well as LipiDex [96] to reduce matrix-based false-positive features. In a third step, lipid annotation can be performed with customizable precursor lists based on databases containing lists derived experimentally or using computational methods, such as LIPID MAPS [100], LipidWeb (formerly LipidHome) [101], or Human Metabolome Database [102] (HMDB), which are sources of in-silico generated and/or experimentally detected lipids. Up to the point that the precursor MS is annotated, there is no relevant difference between lipidomics and metabolomics approaches. All of these software packages are able to annotate lipid species at the precursor MS level, but MS/MS spectral annotation has also become indispensable for (i) differentiating between coeluting isobaric lipids in order to correctly identify the different lipids at the lipid species annotation level (e.g., PC 38:4), (ii) obtaining deeper structural insights that are useful for identifying fatty acyl/alkyl chains (e.g., PC 18:0_20:4), and (iii) differentiating between coeluting regioisomers (e.g., PC 18:0/18:2, PC 18:2/18:0). There are several approaches that can be employed for the MS/MS-based identification and annotation of lipids. LipidMS [95], LipidMatch [86], LDA 2 [54], and LipidIMMS [103] apply customizable fragmentation rules (*m/z*, intensity) to identify various lipid classes, acyl/alkyl chains, and potential *sn*-1 and *sn*-2 positions according to specific fragment intensities. Rule-based methods work well, and can be devised manually based on in-house experiments and the literature. The drawback of rule-based methods is the effort required to implement adequate rules, because the type of mass spectrometer used, the fragmentation techniques (CID, HCD) applied, and the collision energy must be considered. Furthermore, these methods cannot be applied to a novel class of targets. Another approach, which is used by LipidDex and MS-Dial, is spectral similarity matching through modified dot product algorithms. MS/MS fragmentations are compared with libraries of experimental and/or computationally generated MS/MS data such as the NIST Tandem Mass Spectral Library, MassBank [104], Massbank of North America (MoNa), LipidBlast [105], and Library Forge [106]. LIQUID uses a trained scoring model based on the probability that a specific fragment will be observed at a given intensity [89]. Greazy/LipidLama offers a scoring model based on an identification approach with an additional false-discovery rate function [90] that can simplify manual verification.

The lack of experimentally obtained reference spectra for tandem mass spectrometry causes problems when attempting to identify unknown analytes or those with low concentrations. A number of software tools called in-silico fragmenters that employ a more computational approach have been developed to address this issue over the last decade. MetFrag2.2 [107] combines database searching and fragmentation prediction to identify small molecules from MS and MS/MS data. Reference data from PubChem, ChemSpider, the Kyoto Encyclopedia of Genes and Genomes (KEGG), and offline databases are supported. After performing combinatorial fragmentation in silico, candidates can be filtered and scored with additional criteria such as retention time information, substructures, elements, and reference information. Another fragmenter, (Compound Structure Identification):FingerID” (CSI:FingerID), uses support vector machines (SVMs) and predicts the presence of functional fragments and groups based on provided MS spectra or compounds. These spectral fingerprints and MS/MS fragmentation trees are used to rank the unknown compound [108]. Competitive Fragmentation Modeling–ID (CFM-ID) is used to predict ESI-MS/MS and electron ionization (EI)-MS spectra using in-silico fragmentation techniques. A reference MS/MS spectral library containing over 51,000 known compounds with sets of different collision energies was generated based on information from KEGG and HMBD and used to predict unknown molecules [109]. Due to poor processing performance for large segmented molecules such as lipids, rule-based fragmentation was implemented in CFM-ID version 3.0 to predict the MS/MS spectra of 21 lipid classes, as well as a new scoring system and a chemical classification algorithm. These changes allow predicted ESI-MS/MS spectra of lipids to be obtained 200 times faster and ten times more accurately than when CFM-ID version 2.0 is used [110]. Thus, in-silico mass spectrometry is a fast-growing field, which should lead to advances in novel compound prediction and identification. The steady growth of databases and improvements to algorithms would also be expected to enhance in-silico prediction accuracy in the future.

The quantification of experimental data is another important aspect of lipidomics studies. Due to the lack of available isotopically labeled reference materials and the large number of analytes, only relative (not absolute) quantification is possible in most cases. This means that an amount of analyte is compared to a defined amount of a reference analyte. Most lipidomics studies involve the comparison of different biological groups, so it can be argued that absolute quantities are not relevant, only differences between the groups. However, in our opinion it is important, if possible, to quantify or to normalize lipids in order to compensate for ion suppression and ionization efficiency, as this allows data from studies with different experimental conditions and MS devices to be compared.

Among the LC-MS/MS programs mentioned above (apart from the commercial Lipidomics software solution), only LDA 2 and LipidMatch Normalizer [111] (LMN) are designed to allow comprehensive automated lipid quantification or relative quantification. LDA 2 provides a GUI and is optimized for DDA methods. Excel-based precursor lists with optional retention time settings are used to annotate lipid species at the MS1 level [98], and rule-based decisions are applied at the MS/MS level to confirm the annotation [54]. The software comes with preadjusted parameters for various MS devices and tested rules for several lipid classes, and is easy to customize and expand according to individual requirements. In order to facilitate user control of peak integration and annotation, LDA 2 permits the visualization of precursor MS1 and MS/MS spectra for easy manual verification and direct rule adaptation. LDA 2 can use a sophisticated algorithm to select the most appropriate standard for quantification among the multiple standards available for each lipid class [98]. This procedure minimizes ionization differences caused by the use of gradient elution in liquid chromatography. In contrast to the “all-in-one” approach of LDA 2, LMN can be used in combination with any peak-picking and annotation software such as MS-DIAL [88], XCMS [112], or MZmine [91]. There is also a GUI-based solution that integrates MSConvert, MZmine 2, and LipidMatch, called LipidMatch Flow. LipidMatch Flow requires a few extracted blanks and pooled quality control (QC) samples that are representative of all biological samples in order to exclude matrix-based features and create a target list, thus enhancing the peak integration time. Quantification with LipidMatch Normalizer focuses on a scoring system to assign the best lipid standard to the analytes of interest. Therefore, it is important to carefully select relevant standards for each lipid class.

## Batch normalization strategies

Untargeted lipidomic studies often include hundreds to thousands of samples. Depending on the approach, experimental measurements can often take a long time, and may even be interrupted. Therefore, systematic errors can creep in to such measurements due to, for example, time-dependent drift in the instrumental sensitivity, batch-dependent effects such as changes in pH and concentrations in solvents, or slight temperature fluctuations. Ignoring such errors reduces the statistical power of the search for significant differences between the detected analytes and the associated phenotype [113]. There are multiple sample normalization strategies [114]; the most popular utilize internal standard-based normalization or QC-based normalization. The internal standard-based normalization methods suppress systematic errors by using internal standards to perform corrections. Several different software tools with different approaches are available for internal standard-based normalization, such as B-MIS [115], NOMIS [116], and CCMN [117]. As noted above, such methods have been integrated into LDA 2 and LipidMatch Normalizer. The other popular method is normalization based on QCs. These QCs are usually pooled biological samples containing all the characteristics of the biological sample under investigation. QC measurements are performed approximately every tenth sample and are used to normalize the data. There are various methods that can be employed for QC-based normalization, such as Batch Normalizer (based on LOESS regression) [118], StatTarget (a Vectro-machine-based normalization method) [119], and the systematic error removal using random forest (SERRF [120]) technique.

## Shotgun software

Shotgun lipidomics can enable faster sample acquisition than LC-MS/MS lipidomics without the need for time-consuming chromatography (e.g., < 3 min [32]), and offers a very robust basis for quantification due to its stable ionization environment. The effect of shotgun lipidomics from a computational point of view is that much more information is collected simultaneously, as the entire lipid extract is ionized at once. This allows multiple injections of samples to be performed for technical replicas and/or to explore the effects of different experimental conditions. Known problems with lipid aggregation and ion suppression can be reduced by using small sample volumes and concentrations [121]. Multiple sample acquisition through a multiplexed extraction approach [122] and intrasource separation [123] (e.g., negative/positive ionization, base addition, or sample derivatization) enhances ionization efficiency for different lipid classes on their chemical and physical properties [123]. Therefore, the computational challenges are even greater than those associated with liquid chromatographic methods. In recent years, some highly specialized software tools have been developed to process shotgun lipidomics data. MDMS-SL (multidimensional mass spectrometry-based shotgun lipidomics) is an automated identification and quantification approach based on array analysis [124]. The AMDMS-SL libraries are based on “building blocks” that represent basic structures of lipid species, i.e., the head group, the backbone, and the chains. The open source software LipidXplorer [125] was developed by Shevchenko’s group. Based on the Molecular Fragmentation Query Language (MFQL) [126], this software allows the customizable input of search parameters for individual user targets. The acquired MS/MS spectra are converted to an open MS format, merged, and stored in a representative MasterScan database entry. For lipid annotations, the MasterScan can be screened simultaneously with MFQL queries. This software is platform independent and can be employed in DDA approaches involving high-resolution mass spectrometry and triple quadrupole mass spectrometry used in combination with precursor ion and/or neutral loss scans. Analysis of Lipid Experiments (ALEX) is another GUI-based software framework developed for multiplexed high-resolution shotgun lipidomics data. The framework consists of six modules that perform tasks ranging from data conversion to lipid quantification. One of its key features is a lipid database that stores structural information for 85 classes of lipids that encompass more than 20,000 lipid species [127].

Unlike ALEX and LipidXplorer, LipidHunter can be used for both LC-MS/MS and shotgun lipidomics to identify glycerophospholipids. Lipid identification is based on the MS/MS rules for each lipid class. Neutral loss and product ions can be associated with glycerophospholipid-specific class fragments and fatty acyl chains on a user-defined “white list.” Results can be matched with bulk and elemental composition data derived from LIPID MAPS searches. A graphical report and tabular information are provided for quality control and data processing steps [128].

## Pathway mapping in lipidomics

The aim of lipidomics is not only to detect differential intensity patterns obtained under different experimental conditions, but also their association within metabolic pathways. Wheelock et al. showed a comprehensive overview of several available pathway-mapping software packages and visualization/manipulation toolboxes [129] (e.g., KEGG [130], Cytoscape 3 [131], Ingenuity Pathway Analysis, MetaCore™ [132], and VANTED [133]). Compared to other omics fields, pathway mapping is still in its early stages in lipidomics. KEGG provides several databases of biological systems: KEGG GENES for genes and proteins, KEGG LIGAND for endogenous and exogenous substances, KEGG PATHWAY for molecular diagrams with pathway maps, and KEGG BRITE for curated hierarchies of connections between various biological objects [134]. A drawback of KEGG is that its databases have thus far mainly focused on biosyntheses of different lipid classes, not different lipid species. However, experimental data from lipidomics at the lipid species level are available. Filling this information gap is an ongoing process. The LIPID MAPS consortium demonstrated the integration of lipidomics and transcriptomics data from time-dependent differential treatments of RAW 264.7 cells. The VANTED software package was used to map the reaction network to information from the KEGG databases, the literature, and experimental data [135]. This work showed how lipidomics data can be illustrated in lipid pathways/networks at different concentration levels.

## Data quality and reporting

**Fig. 5:**
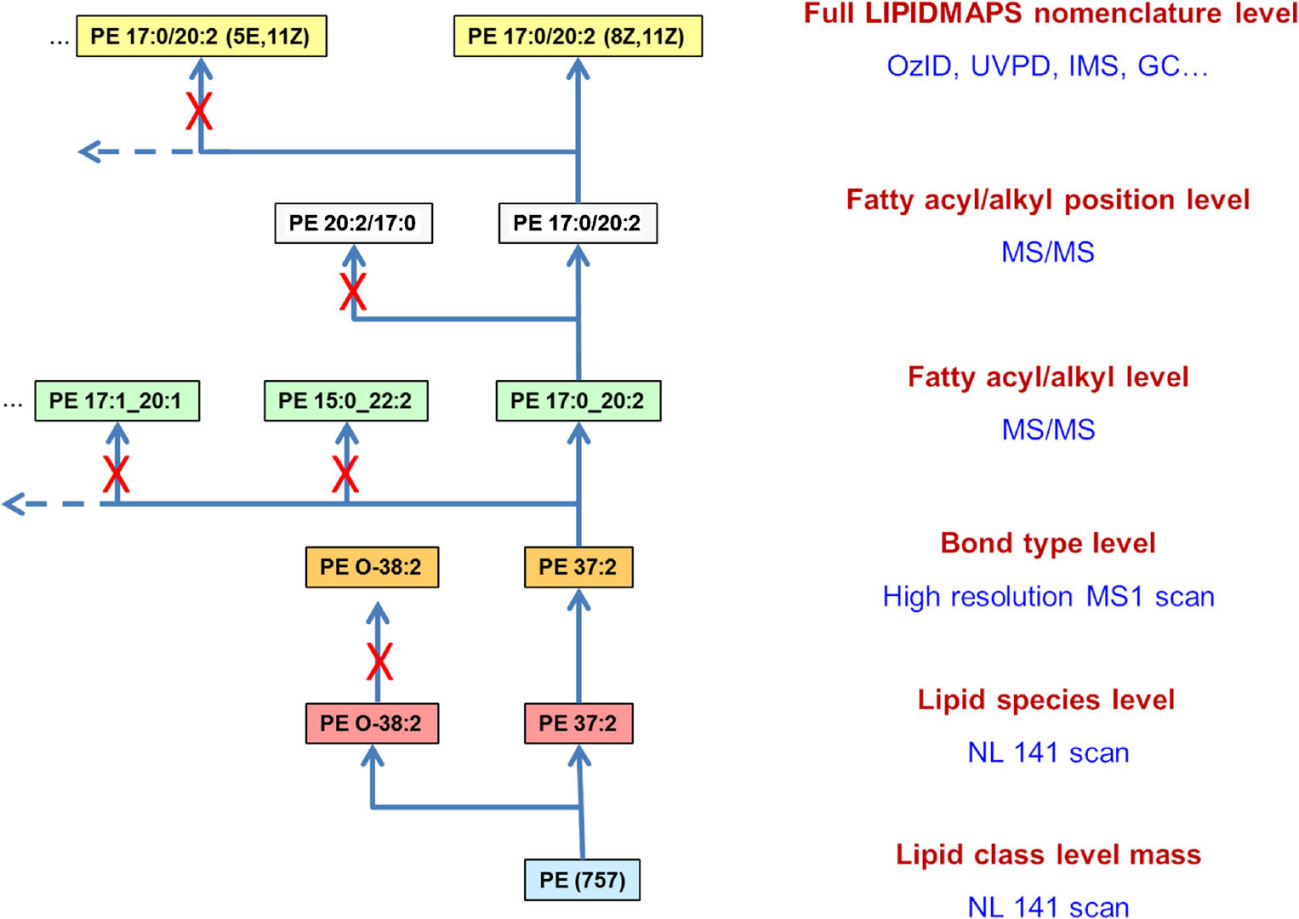
Levels of lipid identification derived from mass spectrometric data, as exemplified by the inherent ambiguities of a phosphatidylethanolamine (PE) species containing an odd-carbon-numbered fatty acyl chain. Various mass spectrometric techniques yield different levels of certainty, which in turn should be reflected in the annotation

In 2005, the International Lipids Classification and Nomenclature Committee (ILCNC) devised the first comprehensive classification system for lipids, which proved to be a hallmark endeavor for the field [1] (Fig. 1). In parallel, the LIPID MAPS consortium established the LMSD, which is the most comprehensive compilation of annotated lipids to date: it contains over 43,000 curated and computationally generated lipid species [136]. Based on these fundamental efforts, the shorthand nomenclature for lipids proposed by Liebisch et al. takes into account the MS-derived knowledge about a specific compound [30]. The most important take-home message in this respect is to report only what is unambiguously experimentally proven. For example, a PC at *m/z* 760 can only be reported as PC 16:0_18:1 when the correct fragments for the head group and both fatty acyl residues have been detected. Thus, according to the LMSD (e.g., PC 16:0/18:1(9*Z*)), the annotation of lipid species is only possible when the headgroup, the *sn* positions of fatty acyls, the double-bond positions, and the conformations of the double bonds are known, which is usually not the case. Therefore, the proposed shorthand nomenclature fills the gap in reported standards for ‘partially’ determined lipid species with more than one possibly possible isomers. Returning to the example depicted in Fig. 5, a neutral loss scan with a triple quadrupole instrument would allow annotation at the lipid class level mass; the addition of either chromatography or high mass resolution instrumentation could potentially separate diacyl and ether PE species; MS/MS spectra could confirm the headgroup, the constituent fatty acyls (the fatty acyl/alkyl level), and even their regioisomeric positions (the fatty acyl/alkyl position level); and the use of OzID, UVPD, IMS, or GC could potentially even elucidate the positions and conformations of double bonds. In summary, these classification and nomenclature standards provide the foundations for moving the field of lipidomics forward, as they enable a unified language and unified reporting standards for the global exchange of data in large-scale research consortia. In line with this, the recently founded International Lipidomics Society (ILS) is participating in joint efforts focusing on the production of further guidelines on standardization, data quality assessment, and clinical applications such as the Lipidomics Standards Initiative (LSI), the Plasma Lipidomics Reference Values Group, or method-harmonizing ring trials [137, 138].

Another factor that deserves attention in relation to data quality is the in-source fragmentation of certain lipids that can mimic other naturally occurring lipids, resulting in misannotation. Typical examples would be the demethylation of PC species, which falsely points to the existence of a phosphatidylethanolamine (PE) species; the hydrolysis of glycerophospholipids, which falsely implies the existence of lysophospholipids; or the loss of glycerophospholipid headgroups, which falsely indicates the existence of PA [139]. One simple way to spot these artefacts is to use chromatography, because when (for example) PC 34:1 and LPC 16:0 have the same retention time, the latter must be an in-source fragmented product of the former. However, this type of artefact can be avoided by carefully choosing and validating the ion source parameters [139].

## Conclusion

With the development of new analytical methods and workflows, it is becoming increasingly conceivable that the determination of lipidomes of whole organisms on a molecular basis will become feasible. This would have a tremendous impact on the evolution of lipidomics, as it would provide far greater insight into the underlying functional roles of lipids. Nevertheless, there are certain prerequisites that must be met before such an ambitious objective can be achieved. The first and most important ingredient is data processing; although progress has been made in this area over the last decade, we are still waiting for fully automated software solutions such as those used in proteomics, which would be particularly useful for probing structural features in detail at the molecular level. The second major issue is quantification, which is most often inherently linked to the nonquantitative nature of electrospray. While relative values are sufficient when comparing statistical groups, methods that can deliver reliable quantitative values will be needed if lipidomics is to develop into a field that can be applied in clinical diagnosis and eventually even for prognosis. These issues are, however, the focus of a great deal of ongoing research. If these problems with annotation, standardization, automation, and quantification can be overcome, the full potential of lipidomics will be unleashed for functional and clinical studies.
